# Prospective clinical implementation of optional implant treatment into pregraduate dental education—mini implants for retention and support of mandibular overdentures

**DOI:** 10.1186/s40729-021-00371-6

**Published:** 2021-09-10

**Authors:** Peer W. Kämmerer, Jens M. Wolf, Ingo Buttchereit, Bernhard Frerich, Peter Ottl

**Affiliations:** 1 Department of Oral, Maxillofacial and Facial Plastic Surgery, University Medical Centre Mainz, Augustusplatz 2, 55131 Mainz, Germany; 2 Department of Oral, Maxillofacial and Facial Plastic Surgery, University Medical Centre Rostock, Schillingallee 35, 18057 Rostock, Germany; 3 Department of Prosthodontics and Materials Science, University Medical Centre Rostock, Strempelstraße 13, 18057 Rostock, Germany; 4 Department Life, Light & Matter, University of Rostock, 18051 Rostock, Germany

**Keywords:** Mini implants, Student education, Undergraduate dental curriculum, Implant surgery, Implant prosthodontics

## Abstract

**Background:**

The primary aim of the study was to evaluate mini implant (MDI) survival, prosthodontic maintenance, and patient-reported outcome measures after conducting an optional pregraduate academic course on patients with mandibular edentulism including fabrication of overdentures and MDI planning and placement as well as chair-side incorporation of the respective restauration. In a prospective clinical study, 20 patients received 80 MDIs that were restored with mandibular overdentures. All treatment steps including placement of 40 MDIs were conducted by undergraduate students under strict guidance of a consultant. Next to students’ perceptions after participation, survival of MDIs, and prosthodontic maintenance, patients’ perceptions as well as peri-implant parameters were assessed after 4 weeks and 3 and 12 months.

**Results:**

Three MDIs fractured (two during insertion and one after 3 months; total survival 96.25%). Two overdentures fractured and a total of 23 cases of minor prosthodontic maintenance were required. Over time, patients’ satisfaction significantly increased. Besides, questionnaires showed a high rate of students’ positive perception and high self-confidence to include MDI therapy into own practice.

**Conclusions:**

The results are in accordance to those reported by postgraduate dentists. In accordance, therapy with MDI-retained mandibular overdentures seems to be feasible and successful at pregraduate level if the students receive guidance.

## Background

Dental implant therapy (DIT) needs proper knowledge and training that should be implemented into dental academic education. In accordance, DIT is mostly prevalent during the last 2 years of academic training [1]. There is a variety of programs and methods relating to preclinical DIT such as jaw models and drilling simulators [2, 3], but direct patient care remains the most beneficial instructional method [1]. Though, even if DIT has been incorporated into most pregraduate academic dental institutions in the USA and Europe, it has been taught markedly conservative when compared to other treatment modalities [1]. Nevertheless, there is evidence that dental implants that were provided by pregraduate students (undergraduate students in the clinical part at dental school)—under work standardization, strict guidance, and proper patient selection—show high survival and success rates, even in the esthetic zone [4–6]. Also, implementation of implant-restorative programs in undergraduate dental curricula resulted in a high students’ satisfaction [7]. Besides, single-tooth implant and implant overdenture therapies promote the standard of care at general dentist level and are described to be feasible at pregraduate level as well [8].

Implant-supported overdentures are considered to be the treatment of choice for rehabilitation of the edentulous mandible [9, 10]. Even so, standard implant placement protocols often need bone grafting procedures before implant insertion. Here, chronic systemic diseases and medications such as anticoagulation may hamper advanced surgical methods [11, 12]. Together with a certain healing period before prosthetic loading, placement of conventional implants may also increase patients’ morbidity and discomfort [13]. Even if the considerable costs of standard-width implants are no issue, especially older patients refuse to undergo implant placement procedures due to anticipated pain and associated complications [14]. Mini dental implants (MDIs; ≤ 2.5 mm diameter [15]) for immediate loading of a mandibular overdenture offer a less invasive and cost-effective alternative to standard-width implants [16–18]. They may be indicated in patients dissatisfied with conventional overdentures and with limitations or contraindications for placement of conventional implants. In a recent systematic review, Lemos et al. analyzed the results of 2330 MDIs in the lower and 164 MDIs in the upper jaw. Here, a cumulative survival of 92% after 6–7 years (significant more failures in the maxilla when compared to the mandible) could be shown. Survival values of MDI > 10 mm in length were similar to those obtained by standard implants. In the mandible, the reported peri-implant bone loss accounted for less than 1.5 mm. In terms of patient-reported oral health-related quality of life, a significant increase in terms of retention, stability, chewing, speaking, comfort, esthetics, satisfaction, and social life/quality of life was proven [19]. For retention of mandibular overdentures, four MDIs are commonly used, showing higher rates of success and masticatory ability when compared to less MDIs [19, 20]. For four implants supporting mandibular overdentures, a 1-year survival rate of 96–97% [16, 21] and of 96% after 3 years [22] was seen. However, restauration of implant overdentures via mandibular overdentures is included in a paucity of predoctoral programs only [23], and there is no report on planning and insertion of MDIs as well as providing MDI-stabilized overdentures by dental students in the literature yet.

Therefore, the aim of the study was to develop and evaluate an optional pregraduate, academic teaching course including fabrication of overdentures and MDI planning and placement as well as chair-side incorporation of the respective restauration. As patient-related outcomes are of pivotal importance and receive high emphasis in health care assessment, primary research parameter was implant survival and prosthodontic maintenance. Secondary parameters of interest were students’ as well as patients’ perceptions before and after the course as well as other implant related data.

## Methods

### Patients

In a prospective clinical study, 20 patients were included. All patients voluntarily took part and participated in the student Prosthodontics course at the University Medicine Centre Rostock, Germany. Next to an edentulous mandible, inclusion criteria were a mandibular overdenture with insufficient adjustment due to anatomical reasons together with a minimal mandibular height of 13 mm and a minimal mandibular diameter of 3 mm together with a healthy mucosa. All patients declared written consent, and the study was approved by the local ethical committee of the University Center Rostock, Germany (A 2014-0175). The exclusion criteria included poor oral hygiene and untreated periodontal disease in the opposing arch, lack of compliance, severe metabolic disease, immunosuppressive therapy, and pregnancy or nursing. All prosthodontic and surgical procedures were conducted by dental last year students under strict guidance of a consultant of the Department of Prosthodontics and Materials Science (JMW) and a consultant of the Department of Oral, Maxillofacial and Facial Plastic Surgery (PWK, IB).

### Students’ assessment before participation

Before participation to the course, each student was asked for age, gender, existing knowledge in terms of implant surgery, and implant prosthodontics (structured into “practical,” “theoretical,” “insufficient,” and “none”). Using an open question, students were also asked for the main reason of participation. Before inclusion into the study, students introduced potential study candidates from the student prosthetics course to the consultant and the patients were included if suitable.

### Patients’ assessment before treatment

The following data was collected using a structured questionnaire:

- Age, gender,
- Duration of mandibular edentulousness, age and subjective fitting of the last overdenture (categorized into “very good,” “good,” “acceptable,” and “unsatisfactory”),
- Reasons no implants were planned so far and
- Concomitant diseases including medication.

Next, the following data were collected from the dentist/student:

- Objective fitting of the existing overdenture and
- Height (mm; from panoramic x-rays) of alveolar bone in the interforaminal area

**Fig. 1:**
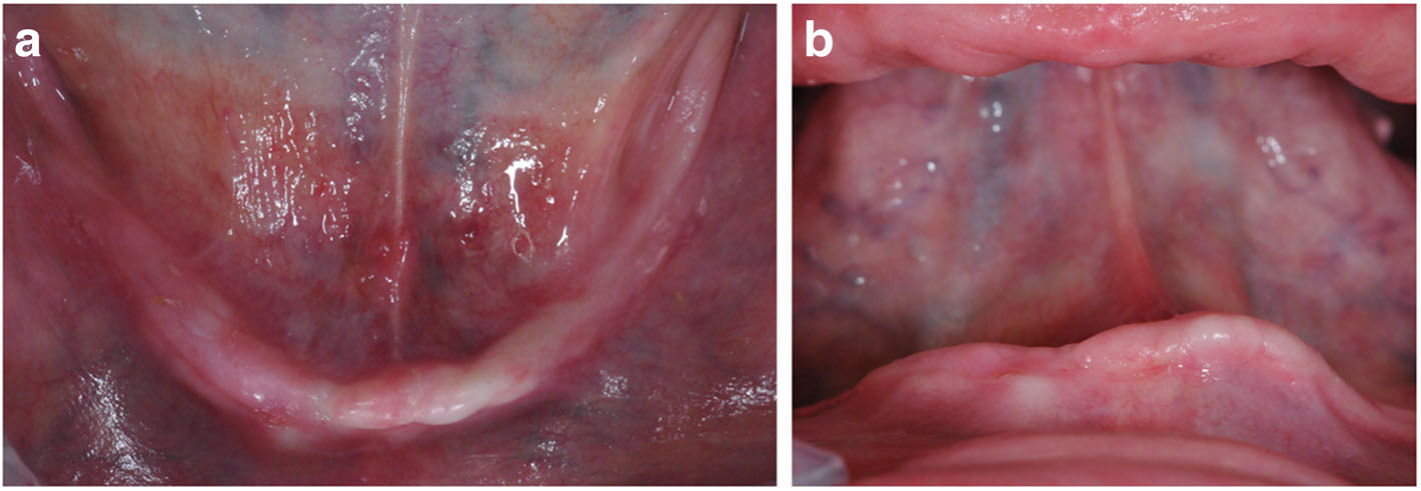
**a**, **b** Exemplary preoperative photographs from the top (**a**) and from the front (**b**)

**Fig. 2:**
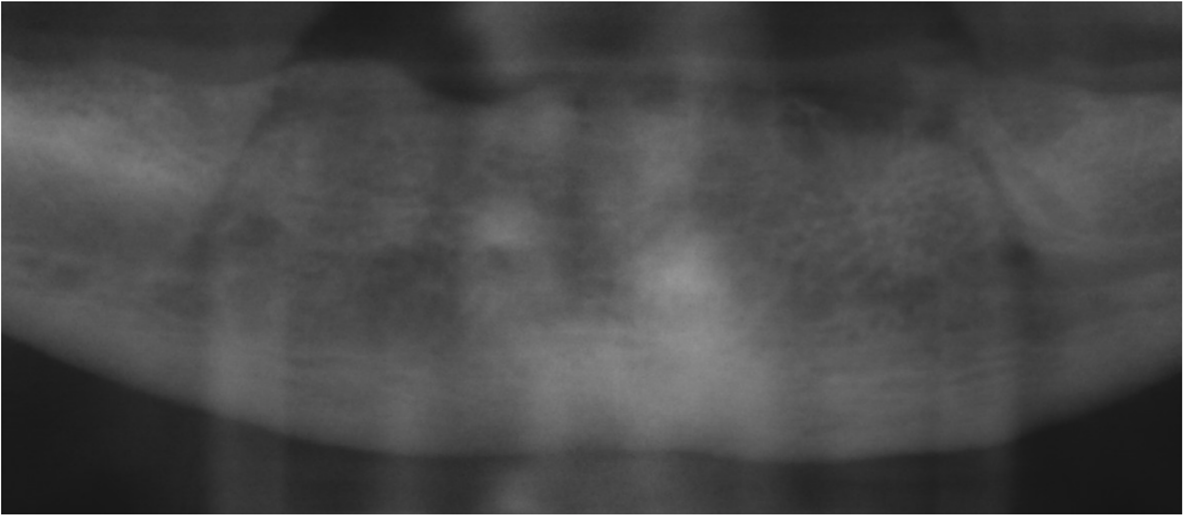
Excerpt from an exemplary preoperative radiography showing the mandibular bone with sufficient height

For treatment, the following procedures were carried out:

- Photo documentation (Fig. 1a, b),
- Conventional radiography (orthopantomography; Fig. 2),
- Impressions for situation models and
- Completion of the OHIP-G 14 questionnaire.

### Treatment

#### Prosthodontic procedures before surgery

In all cases, new mandibular overdentures were fabricated by dental last year students under supervision of a consultant. In brief, preliminary impression of the edentulous jaw was made with Schreinemakers impression trays and alginate (HS-Maxima®, Henry Schein, Langen, Germany). Using the resulting cast model (Implantat-rock®, Picodent, Wipperfürth, Germany), an individual tray (Impression Tray Resin LC, Henry Schein) was manufactured. Border molding was performed dorsal and sublingual with impression compound (Impression Compound®, Kerr, Biberach, Germany) and ISO Functional Sticks (GC Corporation, Tokyo, Japan). The final functional impression was made with a polyvinyl siloxane (Coltex extrafine, Coltene, Altstätten, Switzerland). For determination of vertical height, bite rims were used. After setting up the teeth (Artegral®, Merz Dental, Lütjenburg, Germany), the wax fitting of the dentures was carried out. Afterwards, the fabricated denture was inserted.

#### Surgery

**Fig. 3:**
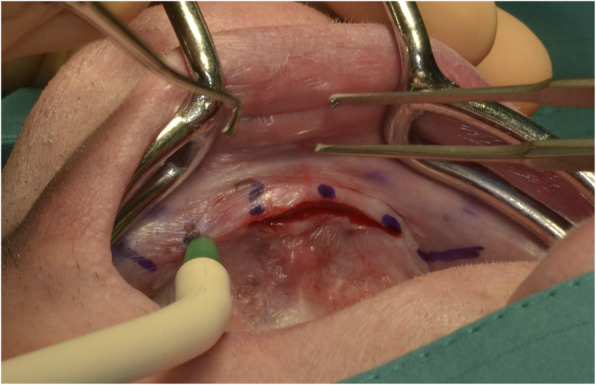
After marking of the planned MDI-positions, a mucoperiosteal flap without releasing incisions is raised

**Fig. 4:**
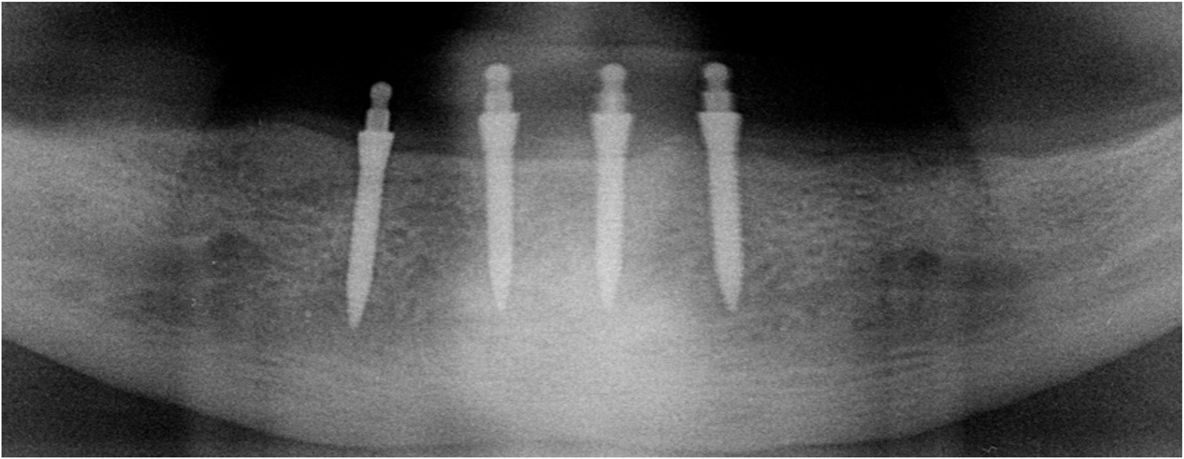
Excerpt from an exemplary preoperative radiography showing inserted MDIs

After prosthodontic preparations, all participants received 4 intraforaminal MDIs (3M, Seefeld, Germany) with a diameter of 1.8–2.2 mm and a length of 13–15 mm as described in the literature [24]. The first 2 implants were inserted by a consultant and the other 2 by a dental last year student. In brief, the insertion points were marked on dry gingiva. Afterwards, infiltration anesthesia (4% articaine with 1:400 000 epinephrine (3M, Seefeld, Germany) or 4% articaine without epinephrine (Sanofi, Berlin; for patients with comorbidities)) was applied. Using a 15C scalpel, the summit of the mandibular ridge was perforated corresponding to each marked point and a small mucoperiosteal flap without vertical release incision was raised (Fig. 3). The pilot drill (1.1 mm Pilot Drill, 3M, Seefeld, Germany) was inserted lightly until perforation of the cortical plate (1/3 of the implant length). Now, the planned implant was inserted using a driver (Winged Thumb Wrench, 3M, Seefeld, Germany). For achieving parallelism, the first implant was screwed in ½ way before the second hole was drilled in order to use each implant as placement guide for the next. Each implant was inserted until the abutment head protruded for the soft tissue without any thread portions visible (Fig. 4). If the primary stability deemed too high, the implant was carefully removed, the pilot drill was inserted lightly till ½ of the implant length, and the implant was re-placed.

#### Prosthodontic procedures after surgery

**Fig. 5:**
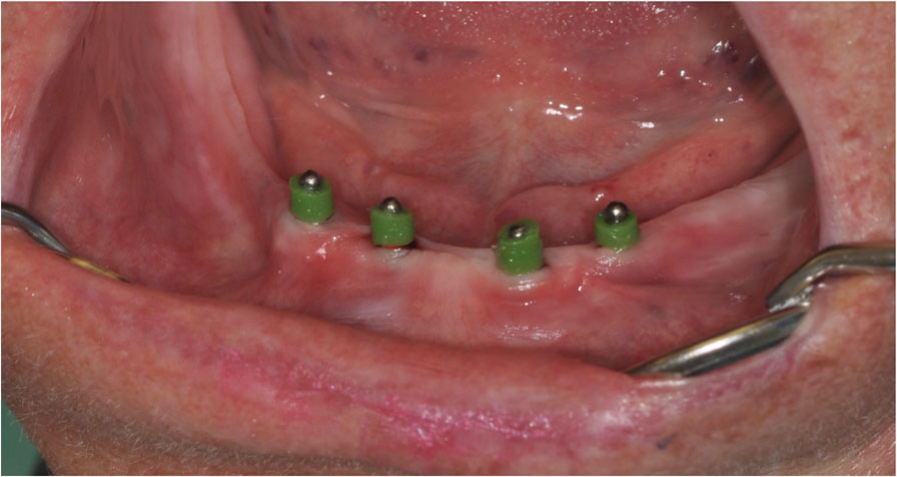
Blocking of undercut spaces of the implants with rubber tube before insertion of the pre-manufactured overdenture

**Fig. 6:**
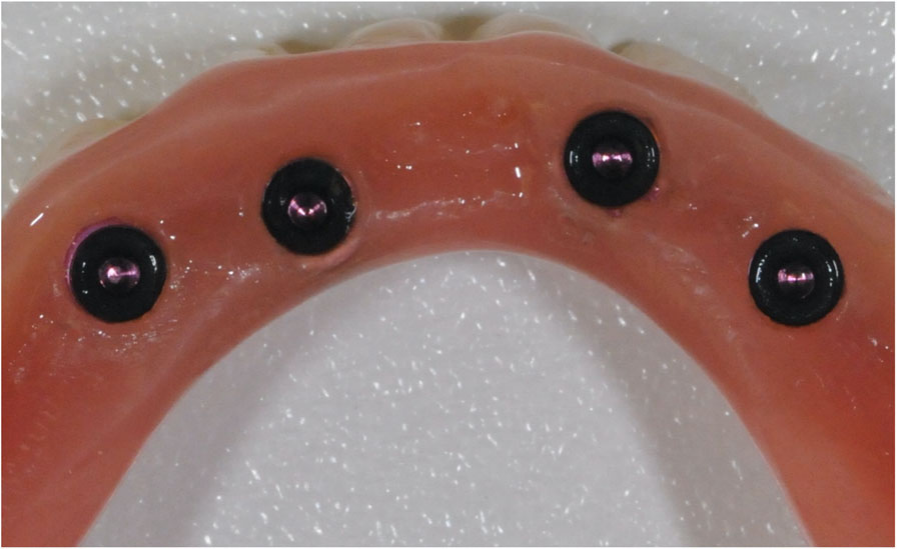
Metallic housings retained into the overdenture

If the insertion torque of at least 1 implant was < 35 Ncm, a delayed loading protocol was chosen. In these cases, after insertion of the MDIs, the overdenture was relined with a soft material (Secure Soft Reline®, 3M ESPE) and the soft material was replaced after 4 months. For immediate loading (≥ 35 Ncm for all implants) as well as after the delayed loading time period, 2 mm of a rubber tube (Blockout Shim, 3M, Seefeld, Germany) was used to block the undercut spaces of each implant (Fig. 5). Afterwards, a hole for the metal housings was drilled in the inner surface of the overdentures and cold curing acrylic was used to underline and retain the metal housings as described in the literature (Fig. 6) [25]. After metal housing installation, the frontal border of the denture was shortened 1–2 mm from canine to canine.

### Students’ assessment after participation

Via structured questionnaire, students were asked via open and closed questions whether:

- They deemed the treatment to be suitable (yes/no),
- They were satisfied with the support of the lecturers (yes/no),
- They would dare to undertake such a treatment (yes/no),
- They think that the course has increased their knowledge (yes/no),
- They would participate other such learning opportunities (yes/no),
- The course was worth it (yes/no),
- They can imagine such therapies in own practice (yes/no) and if
- They would recommend the course (yes/no).

### Patients’ assessment after treatment

#### Follow-up examinations

All follow-up examinations were conducted by the consultants participating in the course. At 4 weeks (T1) and 3 (T2) and 12 months (T3) after implant therapy, the following parameters were collected:

*Implant-related data*:

- Survival (yes/no),
- Bleeding on probing (BOP; yes/no),

*Overdenture-related data*:

- Need of maintenance (fracture, relining, change of retention rings).

*Patient-related data*:

- OHIP-G 14.

### Statistics

Raw data sets were saved in Excel® sheets (Microsoft Corporation, Redmond, USA) and subsequently transferred into SPSS Statistics® (version 23.0.0.2, MacOS X; SPSS Inc., IBM Corporation, Armonk, NY, USA). Data were expresses as mean, standard deviation (SD±), minimum (min), and maximum (max). Normal distribution was checked using non-parametric Kolmogorov-Smirnov test, and results were analyzed for statistical significance by the use of analysis of variance (ANOVA), unpaired non-parametric Wilcoxon-Mann-Whitney U test, and Students’ t test.

## Results

### Students’ assessment before participation

Twenty students (female *n* = 14, male *n* = 6; mean age: 27 years (min: 21, max: 33; standard deviation (SD): 3.9) took part in the course. Of those, 9 had theoretical knowledge in implant surgery and 3 in implant prosthodontics. In terms of implant surgery, 7 indicated insufficient and 4 no knowledge at all. For implant prosthodontics, 7 students had insufficient and 10 no knowledge. Eleven students wrote that they participated due to personal interest in dental implant therapy. Five wanted to gain new experience and 4 indicated that they were not satisfied with the treatment options they learned so far.

### Patients’ assessment before treatment

Twenty patients (female *n* = 14, male *n* = 6; mean age 69.6 years (min: 56, max: 87; SD: 8.9) were included. They have been edentulous in the mandible for a mean of 165 months (min 36, max 360; SD 105). All of them had mandibular overdentures that were manufactured at a mean of 35 months (min 1, max 168; SD 48) before participation in the course. Here, due to anatomical reasons, an insufficient adjustment was seen. In accordance, the 8 patients reported the overdenture to be “acceptable” and 12 patients voted for “unsatisfactory.” Ten patients said that they were not informed about dental implants before while 9 patients did not choose implants out of financial reasons and 1 patient was afraid of the surgical procedure. For concomitant diseases, 4 patients suffered from arterial hypertonia, 4 from diabetes mellitus, and 3 from atrial fibrillation. Seven patients were under treatment with inhibitors of platelet aggregation (aspirin *n* = 4, clopidogrel *n* = 3), and 4 patients needed insulin. From an objective point of view, the fitting of 2 of the overdentures was rated to be “good,” 11 to be “acceptable,” and 7 to be “unsatisfactory.” A mean radiographic height of the alveolar bone of 33 mm (min 13, max 50; SD 10) was seen. For OHIP-G 14 survey, a mean value of 21.05 (min 6, max 48; SD 11.7) was assessed.

### Treatment

**Fig. 7:**
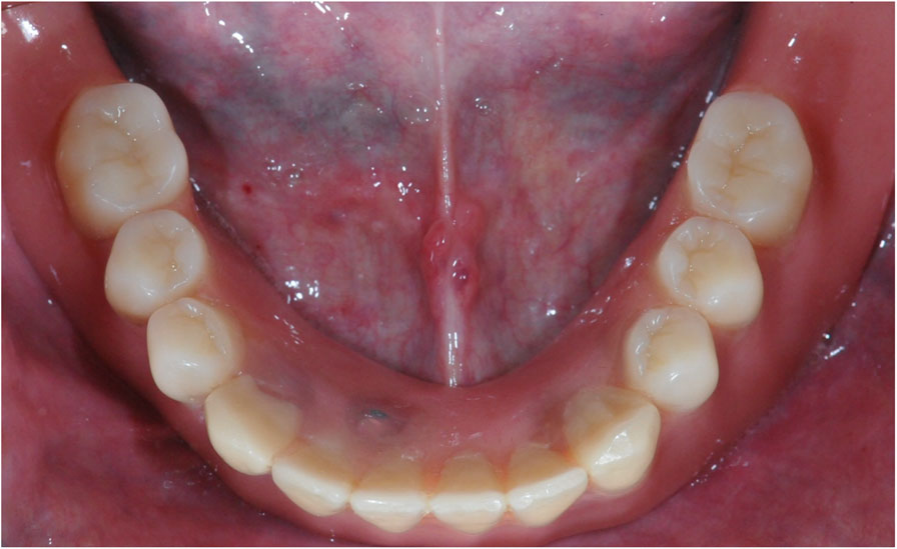
Incorporated overdenture

After manufacturing the new overdentures, 66 MDIs with a diameter of 1.8 mm and 14 MDIs with a diameter of 2.1 mm were inserted. The respective lengths were 13 mm (*n* = 40) and 15 mm (*n* = 44). The mean insertion torque was at of 24 Ncm (min 10, max 45; SD 12). In accordance, 8 cases could be immediately loaded (MH-1 housing *n* = 7, MH-2 housing *n* = 1; Fig. 7), and in 12 cases, a delayed loading protocol was carried out. During implant placement, 2 implants fractured (both 1.8 × 15 mm). One of those was replaced; in the second case, no other implant was inserted.

### Students’ assessment after participation

All students deemed the conducted treatment to be suitable for the selected patients and were satisfied with the academic support. Sixteen students indicated that they would dare to undertake such a treatment while the other 4 would do it again under guidance. The course increased the knowledge in all students (9 students wrote that they learned that simple implantological concepts are an option, 6 learned basics of implant therapy, and 5 students gained knowledge in terms of interdisciplinary concepts). The students voted unanimously that they would participate to other such learning opportunities, that the course was worth it, that they could imagine such therapies in own practice, and that they would recommend the course.

### Patients’ assessment after treatment

#### Follow-up examinations

##### Four weeks (T1)

**Fig. 8:**
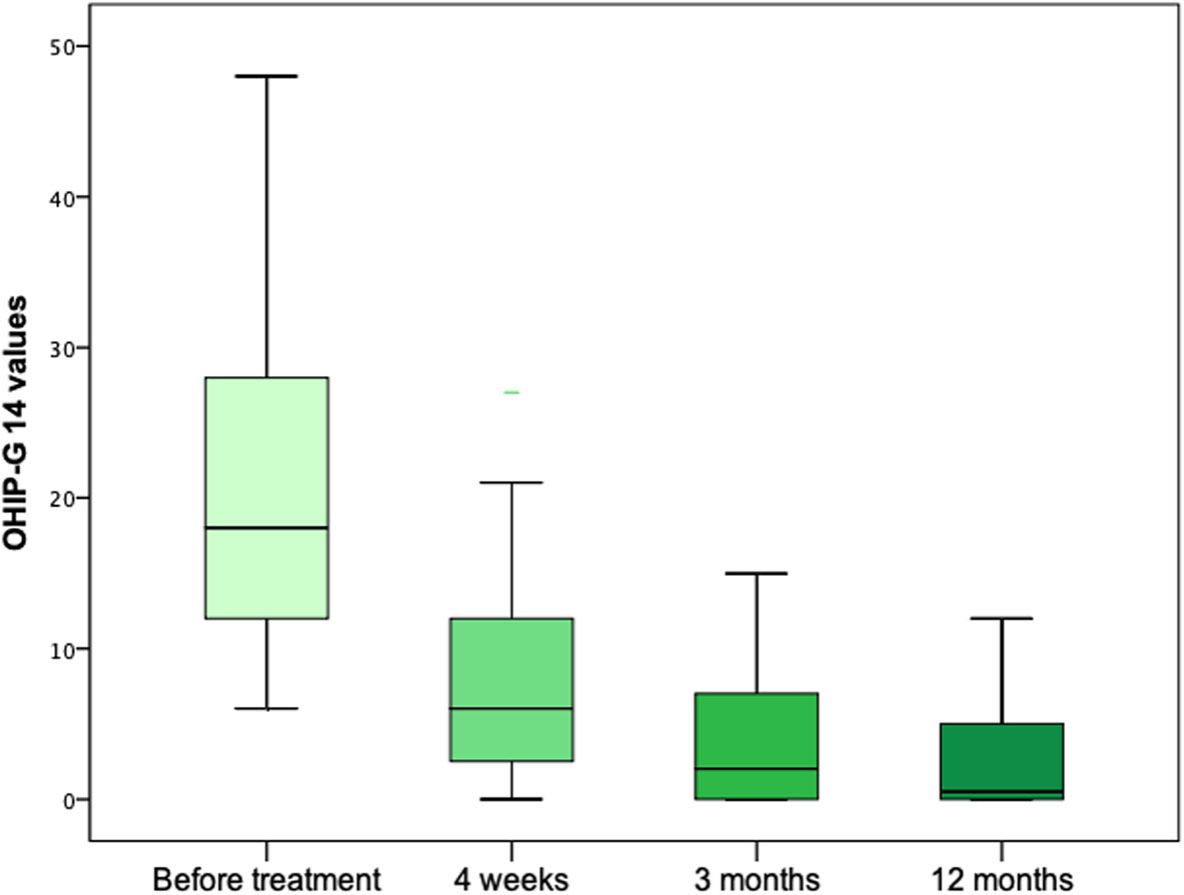
Boxplots showing OHIP-G 14 values of included patients before treatment as well as after 4 weeks, 3 months, and 12 months of follow-up

Seventy-nine of 79 implants were healthy in situ and *n* = 49 (62%) were negative on BOP. In 8 patients, maintenance was needed (cleaning of implants *n* = 7, repair of a fractured overdenture *n* = 1). The mean OHIP-G 14 value was at 9 (min 0, max 27; SD 8.7 *p* < 0.001 when compared to pre-therapeutic levels; Fig. 8).

##### Three months (T2)

After 3 months, another implant was lost from the group with immediate loading resulting in a survival of 98.7% (78/79) respectively in a total survival of 96.25% (77/80). The patient was satisfied with the 3 remaining implants and refused to get another implant. Fifty-two implants (67%) were negative on BOP (*p* > 0.05 when compared to T1). In 11 patients, maintenance was required (relining of overdentures *n* = 6, change of retention rings *n* = 5), and the mean OHIP-G 14 value was at a mean of 4.2 (min 0, max 15; SD 5.3; *p* < 0.001 when compared to pre-therapeutic levels and T1; Fig. 8).

##### Twelve months (T3)

Here, 3 patients with another 12 implants and 3 overdentures could not be included as one patient died and two patients could not be contacted. In accordance, 66 implants and 17 overdentures in 17 patients were evaluated. All implants were healthy in situ and showed a positive BOP in 20 cases (30%; *p* < 0.05 when compared to T1). Maintenance was needed in 6 patients (change of retention rings *n* = 3, relining of overdentures *n* = 2, repair of a fractured overdenture *n* = 1). At this time point, the mean OHIP-G 14 value was 3.17 (min 0; max 12; SD 4.2; *p* < 0.001 when compared to pre-therapeutic levels as well as T1 and T2; Fig. 8).

## Discussions

In dental implant therapy (DIT), lack of experience or technical skills may result in disadvantageous results or even serious complications [2], and there is increasing interest in providing adequate DIT-related education for dental students, even if with variable content and duration [6]. Dental and surgical simulators may allow familiarizing with a variety of procedures before performing them on a patient, but only live procedures on real patients will give the student an overview of the treatment situation. Also, actual clinical outcomes may be the best measurement for assessing overall effectiveness of the respective training [6, 8]. Therefore, the present study evaluated an optional pregraduate DIT-course consisting of simple prosthodontic and surgical tasks. Here, after a follow-up of 1 year, an implant survival of 96.25% was seen. Two implants fractured during insertion and one implant was lost after 3 months. These values are in accordance to the literature on treatments performed by postgraduate residents, and implant loss after 1 year is rarely reported [16, 19, 21, 22, 26, 27], though a high need of prosthodontic maintenance could be seen and it seems that prosthetic complications of MDI-retained overdentures are frequent [27]. In accordance, Lemos et al. summarized a mean overdenture-related survival of 91%, and most fractures occurred in the area of metal housing but could be repaired easily. Interestingly, most of these failures occurred in the maxilla (32% versus 5% in the mandible) [19]. Schwindling and Schwindling also experienced an incidence of overdenture fractures of 24% as well as the need for minor maintenance that should not be under-estimated [28, 29]. In total, maintenance rates in the literature are similar to those reported in the study at hand. It has to be discussed if relining of an implant-retained overdenture or change of retention rings should be interpreted as a complication or rather a part of the treatment concept [30, 31]. In conclusion, the present study supports data obtained by others showing that undergraduates will receive acceptable clinical outcomes of implant-related treatments [32–36] even if data on MDI-related restorations from students—let alone in a prospective clinical study—are not available yet. In cases of MDI, special care should be undertaken at time of implant insertion as they are prone to fracture as described in the literature as well [28, 37]. In accordance, when compared to standard implants, the insertion torque has to be significantly reduced and strict bone-preconditioning via drilling is recommended [28, 38].

In the past, students reported a lack of preparedness in providing routine DIT [26, 39]. This is analog to the present study as the majority of students also deemed themselves to be not prepared for DIT. In brief, 11/20 wrote that they had at least insufficient knowledge on implant surgery and 17/20 that their knowledge was at least insufficient in terms of implant prosthodontics. After participation, all students quoted a gain of practical knowledge by following the course. This is comparable to other pregraduate courses on DIT reported in the literature [7]. Besides, all students thought of carrying out DIT with MDIs in their own private practice. Analog to this, it was reported that graduates who actively participated in formal as well as preclinical and clinical implant education conduct significantly more often DIT at general practice level, i.e., restore significantly more implants and refer more patients to surgical specialists when compared to others with a lack of such an implant curriculum [40, 41]. In accordance, an implant program such as presented in the present study may increase students’ awareness that dental implants are a treatment option and are beneficial for suitable patients.

There are several patient-related factors influencing and defining the characteristics and the number of implants to be inserted. First, the choice of implant strongly depends on the quantity and quality of bone tissue available. Here, in case of a low bone thickness, MDIs are an alternative rehabilitation option for the edentulous mandible. As bone augmentation techniques are not needed and a flapless approach is possible in selected cases, MDI will decrease postoperative morbidity and therefore increase patients’ acceptance [42]. Whereas in most of the studies MDI were inserted in a flapless approach [29, 42], in the present analysis, small flaps were raised due to the narrow alveolar ridges in order to prevent accidental lingual perforation. This bias results in the teaching environment and in order to overcome limitations of the flapless approach such as limited visibility and the lack of water cooling. Besides, Ribero et al. could not find significant differences in terms of pain and postoperative morbidity when comparing flapless and flap technique in cases of standard implants [42].

As expensive specific retention systems are not needed, MDI may also be a good choice for patients with limited finances that do not want to give up the benefits of retained overdentures. Besides, the prosthodontic technique of incorporating an MDI into a pre-existing overdenture is an approach that can be learned easily and seems suitable for pregraduates getting in touch with DIT. Also, the manufacturing of new dentures is mainly based on standard techniques for conventional overdentures.

Lower scores of the Oral Health Impact Profile (OHIP; ranging from 0 to 56) represent a better oral-related quality of life and well-being. In brief, patients’ OHIP-reported satisfaction significantly increased in comparison to the old, non-MDI-supported dentures after 4 weeks and 3 and 12 months such as seen in the literature [20]. This clearly strengthens the weak existing evidence [43] that MDI-retained overdentures achieve favorable results in terms of patient perceptions. In general, it could be shown that OHIP-scores of patients with four MDIs are comparable or even superior with those of patients obtaining overdentures retained by conventional implants [20, 27, 43].

In the literature, bleeding on probing was positive in 33% of implants after three weeks, followed by 33% and 39% after 6 and 12 months [20] that is in accordance to the present study. As a drawback, peri-implant marginal bone level was not measured as routine radiological assessment at follow-up examinations as it was not approved by the local ethics committee.

In the study at hand, not only the prosthodontic part and the planning—as reported by others [4, 7]—but also part of the surgical procedure was conducted by pregraduate students resulting in an interdisciplinary DIT approach in a comparable safe learning environment. Nevertheless, it should be mentioned that such a program needs a high student/teacher relation and therefore a comparable high manpower. For implementation into the dental curriculum, it may also require previous theoretical and laboratory training as well as changes in the course contents and additional resources, instructors with specific expertise, and a long-term patient support structure. In accordance, a necessary change of academic dental curricula towards dental implant therapy may be a demanding and long process that sometimes does not meet the current pace of innovation [23]. In the past, directors of predoctoral programs on dental implant therapy have been predominantly affiliated with restorative dentistry as DIT is considered to be a restorative treatment approach supported by surgical protocols [1, 8]. Even so, as DIT continues to become more pervasive, an increase of surgical expertise may be predicted [44]. In accordance, the present course was conducted interdisciplinary, being held by a prosthodontic and a surgical university department. Even so, as the present study is a pro-post survey including implant survival, prosthodontic maintenance as well as patient- and student-reported outcome only, its impact on several educational aspects for further integration into the dental pregraduate curriculum may be limited.

## Conclusion

Patient-related outcomes are an interesting and promising way to assess pregraduate training programs rather than assessment of an individual student. The present study gives evidence that dental implant therapy planned and performed by pregraduate students yields comparable results in terms of survival rates as well as patients’ satisfaction to those published in the literature. Besides, the course was unanimously rated as positive, productive, and valuable for the future work. Next to pregraduates’ satisfaction, patients’ perception was positive and even increased over the time of follow-up. Therefore, it may be suggested that the use of this strictly guided incremental learning system will result in not only positive patient-related outcomes but also student-related outcomes.

## Data Availability

The datasets used and/or analyzed during the current study are available from the corresponding author on reasonable request.

## Abbreviations

ANOVA: Analysis of variance
BOP: Bleeding on probing
DIT: Dental implant therapy
MDI: Dental mini implant
OHIP-G: Oral Health Impact Profile – German
SD: Standard deviation

## References

[1] Barwacz CA, Avila-Ortiz G, Allareddy V, Tamegnon M, Hoogeveen K (2015). An overview of U.S. predoctoral dental implant programs and their directors. J Dent Educ..

[2] Kinoshita H, Nagahata M, Takano N, Takemoto S, Matsunaga S, Abe S, Yoshinari M, Kawada E (2016). Development of a drilling simulator for dental implant surgery. J Dent Educ..

[3] Kido H, Yamamoto K, Kakura K, Morinaga K, Matsuura T, Matsunaga T, Shimizu H, Takahashi Y, Sato H, Matsuura M (2009). Students' opinion of a predoctoral implant training program. J Dent Educ..

[4] Taylor EJ, Yuan JC, Lee DJ, Harlow R, Afshari FS, Knoernschild KL (2014). Are predoctoral students able to provide single tooth implant restorations in the maxillary esthetic zone?. J Dent Educ..

[5] Lee DJ, Harlow RE, Yuan JC, Sukotjo C, Knoernschild KL, Campbell SD (2011). Three-year clinical outcomes of implant treatments provided at a predoctoral implant program. Int J Prosthodont..

[6] Al-Sabbagh M, Jenkins DW, de Leeuw R, Nihill P, Robinson FG, Thomas MV (2014). Programmatic assessment of a university-based implant training program using patient-reported outcomes. J Dent Educ..

[7] Fijnheer C, Langhorst FR, Wismeijer D (2016). Evaluation of the implant-related restorative undergraduate programme at ACTA, the Netherlands. Part I: students' satisfaction. Eur J Dent Educ..

[8] Barwacz CA, Avila-Ortiz G, Allareddy V, Tamegnon M, Hoogeveen K (2016). Comparison of Canadian and United States predoctoral dental implant education. J Can Dent Assoc..

[9] Thomason JM, Feine J, Exley C, Moynihan P, Muller F, Naert I (2009). Mandibular two implant-supported overdentures as the first choice standard of care for edentulous patients - the York consensus statement. Br Dent J..

[10] Feine JS, Carlsson GE, Awad MA, Chehade A, Duncan WJ, Gizani S, Head T, Lund JP, MacEntee M, Mericske-Stern R, Mojon P, Morais J, Naert I, Payne AG, Penrod J, Stoker GT, Tawse-Smith A, Taylor TD, Thomason JM, Thomson WM, Wismeijer D (2002). The McGill consensus statement on overdentures. Mandibular two-implant overdentures as first choice standard of care for edentulous patients. Montreal, Quebec, May 24-25, 2002. Int J Oral Maxillofac Implants..

[11] Preoteasa E, Melescanu-Imre M, Preoteasa CT, Marin M, Lerner H (2010). Aspects of oral morphology as decision factors in mini-implant supported overdenture. Rom J Morphol Embryol..

[12] Kämmerer PW, Frerich B, Liese J, Schiegnitz E, Al-Nawas B (2015). Oral surgery during therapy with anticoagulants-a systematic review. Clin Oral Investig..

[13] Clavero J, Lundgren S (2003). Ramus or chin grafts for maxillary sinus inlay and local onlay augmentation: comparison of donor site morbidity and complications. Clin Implant Dent Relat Res..

[14] Ellis JS, Levine A, Bedos C, Mojon P, Rosberger Z, Feine J, Thomason JM (2011). Refusal of implant supported mandibular overdentures by elderly patients. Gerodontology..

[15] Jung RE, Al-Nawas B, Araujo M, Avila-Ortiz G, Barter S, Brodala N (2018). Group 1 ITI Consensus Report: the influence of implant length and design and medications on clinical and patient-reported outcomes. Clin Oral Implants Res..

[16] Griffitts TM, Collins CP, Collins PC (2005). Mini dental implants: an adjunct for retention, stability, and comfort for the edentulous patient. Oral Surg Oral Med Oral Pathol Oral Radiol Endod..

[17] Klein MO, Schiegnitz E, Al-Nawas B (2014). Systematic review on success of narrow-diameter dental implants. J Oral Maxillofac Implants..

[18] Della Vecchia MP, Leles CR, Cunha TR, Ribeiro AB, Sorgini DB, Muglia VA, Reis AC, Albuquerque RF Jr, de Souza RF (2018). Mini-implants for mandibular overdentures: cost-effectiveness analysis alongside a randomized trial. JDR Clin Trans Res..

[19] Lemos CA, Verri FR, Batista VE, Junior JF, Mello CC, Pellizzer EP (2017). Complete overdentures retained by mini implants: a systematic review. J Dent..

[20] de Souza RF, Ribeiro AB, Della Vecchia MP, Costa L, Cunha TR, Reis AC, Albuquerque RF (2015). Mini vs. standard implants for mandibular overdentures: a randomized trial. J Dent Res..

[21] Scepanovic M, Calvo-Guirado JL, Markovic A, Delgardo-Ruiz R, Todorovic A, Milicic B, Misic T (2012). A 1-year prospective cohort study on mandibular overdentures retained by mini dental implants. Eur J Oral Implantol..

[22] Elsyad MA, Gebreel AA, Fouad MM, Elshoukouki AH (2011). The clinical and radiographic outcome of immediately loaded mini implants supporting a mandibular overdenture. A 3-year prospective study. J Oral Rehabil..

[23] Ben-Gal G, Ziv Y, Weiss EI, Zabrovsky A (2017). Teaching mandibular implant-supported overdentures in dental schools in North America - a survey. Eur J Dent Educ..

[24] Kanazawa M, Feine J, Esfandiari S (2017). Clinical guidelines and procedures for provision of mandibular overdentures on 4 mini-dental implants. J Prosthet Dent..

[25] Scepanovic M, Todorovic A, Markovic A, Patrnogic V, Milicic B, Moufti AM (2015). Immediately loaded mini dental implants as overdenture retainers: 1-year cohort study of implant stability and peri-implant marginal bone level. Ann Anat..

[26] Chmar JE, Harlow AH, Weaver RG, Valachovic RW (2007). Annual ADEA survey of dental school seniors, 2006 graduating class. J Dent Educ..

[27] Mundt T, Schwahn C, Stark T, Biffar R (2015). Clinical response of edentulous people treated with mini dental implants in nine dental practices. Gerodontology..

[28] Schwindling FS, Schwindling FP (2016). Mini dental implants retaining mandibular overdentures: a dental practice-based retrospective analysis. J Prosthodont Res..

[29] Elsyad MA (2016). Patient satisfaction and prosthetic aspects with mini-implants retained mandibular overdentures. A 5-year prospective study. Clin Oral Implants Res..

[30] Attard NJ, Zarb GA (2004). Long-term treatment outcomes in edentulous patients with implant overdentures: the Toronto study. Int J Prosthodont..

[31] Kleis WK, Kämmerer PW, Hartmann S, Al-Nawas B, Wagner W (2010). A comparison of three different attachment systems for mandibular two-implant overdentures: one-year report. Clin Implant Dent Relat Res..

[32] Maalhagh-Fard A, Nimmo A (2008). Eleven-year report on a predoctoral implant dentistry program. J Prosthodont..

[33] Kronstrom M, McGrath L, Chaytor D (2008). Implant dentistry in the undergraduate dental education program at Dalhousie University. Part 1: clinical outcomes. Int J Prosthodont..

[34] Kroeplin BS, Strub JR (2011). Implant dentistry curriculum in undergraduate education: part 2-program at the Albert-Ludwigs University, Freiburg. Germany. Int J Prosthodont..

[35] Vandeweghe S, Koole S, Younes F, De Coster P, De Bruyn H (2014). Dental implants placed by undergraduate students: clinical outcomes and patients'/students' perceptions. Eur J Dent Educ..

[36] Koole S, De Bruyn H (2014). Contemporary undergraduate implant dentistry education: a systematic review. Eur J Dent Educ..

[37] Hasan I, Bourauel C, Mundt T, Stark H, Heinemann F (2014). Biomechanics and load resistance of small-diameter and mini dental implants: a review of literature. Biomed Tech (Berl)..

[38] Bidra AS, Almas K (2013). Mini implants for definitive prosthodontic treatment: a systematic review. J Prosthet Dent..

[39] Weaver RG, Chmar JE, Haden NK, Valachovic RW (2005). Annual ADEA Survey of Dental School Seniors: 2004 Graduating Class. J Dent Educ..

[40] Yuan JC, Kaste LM, Lee DJ, Harlow RF, Knoernschild KL, Campbell SD (2011). Dental student perceptions of predoctoral implant education and plans for providing implant treatment. J Dent Educ..

[41] Huebner GR (2002). Evaluation of a predoctoral implant curriculum: does such a program influence graduates' practice patterns?. Int J Oral Maxillofac Implants..

[42] Ribeiro AB, Della Vecchia MP, Cunha TR, Sorgini DB, Dos Reis AC, Muglia VA (2015). Short-term post-operative pain and discomfort following insertion of mini-implants for retaining mandibular overdentures: a randomized controlled trial. J Oral Rehabil..

[43] Sivaramakrishnan G, Sridharan K (2017). Comparison of patient satisfaction with mini-implant versus standard diameter implant overdentures: a systematic review and meta-analysis of randomized controlled trials. Int J Implant Dent..

[44] Zimmermann R, Hendricson WD (2011). Introduction of an implant surgical selective into a predoctoral dental curriculum. J Dent Educ..

